# 1,2-Di-*tert*-butyl­ethane-1,2-diyl bis­(*tert*-butane­sulfinamide)

**DOI:** 10.1107/S1600536808042360

**Published:** 2008-12-20

**Authors:** Xiaoxia Sun, Yu Hu, Congbin Fan, Weihong Xiao

**Affiliations:** a Jiangxi Key Laboratory of Organic Chemistry, Jiangxi Science & Technology Normal University, Nanchang 330013, People’s Republic of China; b Experimental Chemistry Center, Nanchang University, Nanchang 330031, People’s Republic of China

## Abstract

In the title compound, C_18_H_40_N_2_O_2_S_2_, a vicinal diamine derivative, the crystal structure is stabilized by two intra­molecular N—H⋯O hydrogen bonds. The distance between the two kernel chiral C atoms is 1.580 (2) Å.

## Related literature

For details of the preparation, see: Sun *et al.* (2005 ▶). For background to vicinal diamines, see: Roland *et al.* (1999 ▶); Lucet *et al.* (1998 ▶). For related literature, see: Alexakis *et al.* (2000 ▶).
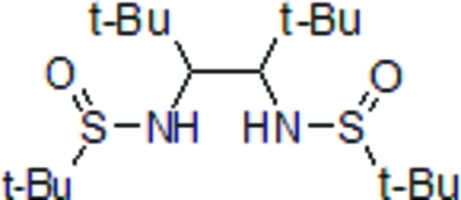


## Experimental

### 

#### Crystal data

C_18_H_40_N_2_O_2_S_2_
*M* *_r_* = 380.64
Monoclinic, 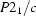

*a* = 13.053 (2) Å
*b* = 9.578 (1) Å
*c* = 18.279 (2) Å
β = 92.069 (8)°
*V* = 2283.6 (6) Å^3^
*Z* = 4
Mo *K*α radiation
μ = 0.25 mm^−1^
*T* = 287 (2) K
0.50 × 0.44 × 0.38 mm

#### Data collection

Bruker P4 diffractometer
Absorption correction: none
4904 measured reflections
4242 independent reflections
3039 reflections with *I* > 2σ(*I*)
*R*_int_ = 0.014
3 standard reflections every 97 reflections intensity decay: 4.6%

#### Refinement

*R*[*F* ^2^ > 2σ(*F* ^2^)] = 0.036
*wR*(*F* ^2^) = 0.096
*S* = 1.00
4242 reflections
238 parameters
2 restraints
H atoms treated by a mixture of independent and constrained refinement
Δρ_max_ = 0.20 e Å^−3^
Δρ_min_ = −0.15 e Å^−3^

### 

Data collection: *XSCANS* (Siemens, 1994 ▶); cell refinement: *XSCANS*; data reduction: *SHELXTL* (Sheldrick, 2008 ▶); program(s) used to solve structure: *SHELXS97* (Sheldrick, 2008 ▶); program(s) used to refine structure: *SHELXL97* (Sheldrick, 2008 ▶); molecular graphics: *SHELXTL*; software used to prepare material for publication: *SHELXTL*.

## Supplementary Material

Crystal structure: contains datablocks I, global. DOI: 10.1107/S1600536808042360/jh2069sup1.cif

Structure factors: contains datablocks I. DOI: 10.1107/S1600536808042360/jh2069Isup2.hkl

Additional supplementary materials:  crystallographic information; 3D view; checkCIF report

## Figures and Tables

**Table 1:** Hydrogen-bond geometry (Å, °)

*D*—H⋯*A*	*D*—H	H⋯*A*	*D*⋯*A*	*D*—H⋯*A*
N1—H1*N*⋯O2	0.850 (9)	2.204 (10)	3.023 (2)	161.9 (16)
N2—H2*N*⋯O1	0.837 (9)	2.210 (10)	3.013 (2)	161.0 (16)

**Fig. 1.**
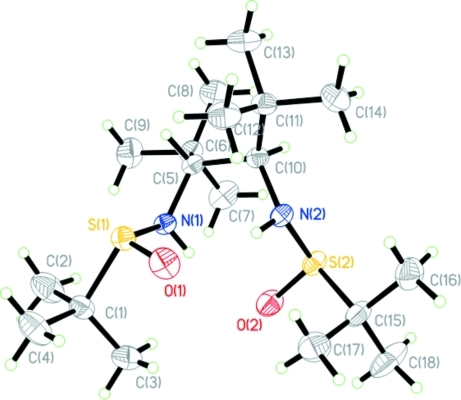
Molecular structure of title compound in the solid state, showing the labeling scheme. The crystallographic 2-fold axis passes through the C5-C10 bond and is perpendicular to the plane of the picture.

**Fig. 2.**
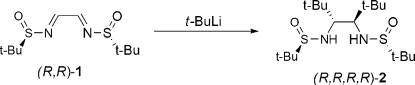
Synthesis of the title compound.

**Fig. 3.**
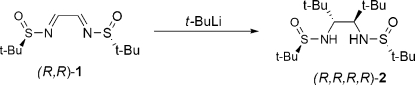
The formation of the title compound.

## supplementary crystallographic information

### Comment

In recent years, enantiopure vicinal diamines have played an increasingly important role in organic chemistry, particularly due to their use as chiral auxiliaries or precursors for the synthesis of a broad family of bidentate ligands (Lucet *et al.*,  1998). Among all organic vicinal diamine compounds, ditertbutyl vicinal diamine are the most promising candidates for those application, mainly due to the great steric hindrance (Roland *et al.*, 1999).

The X-ray crystallographic study confirms the molecular structure previously proposed on the basis of spectroscopic data (Fig. 1). The molecule adopts big steric hindrance with excellent diastereoselectivity and high enantioselectivity.The distance between the two kernel chiral C atoms is 1.580 (2) Å. The syn relative configuration of the newly formed stereocenters is expected according to the Cram rule (Alexakis *et al.*, 2000).

### Experimental

Compound (Ia) was prepared from Bis-[(R)- N-tert-Butanesulfinyl]ethanediimine (264 mg, 1.00 mmol). To a flask was added the Bis-[(R)- N-tert-Butanesulfinyl] ethanediimine in the specified solvent and the solution was then cooled to 195 K under a argon atmosphere. 2 mol/*L* t-BuLi in diethyl ether (2.0 ml) was added slowly to the solution and stirred for 3-5 h. The reaction mixture warmed to room temperature and stirred for a further 2 h. The mixture cooled to 273 K and quenched by the addition of a saturation solution of sodium sulfate. Organic phase separated and aqueous phase extracted with ethyl acetate. Combined organic layers were dried over magnesium sulfate, filtered and concentrated. The residue purified via flash chromatography to afford disulfinamide (Sun *et al.*,  2005).

Finally the colorless crystals were obtained by slow vapour diffusion of diethyl ether. The titile compound was characterized by melting point, Rotation, IR, HRMS and NMR(m.p.134.7–135.4 K). ^1^HNMR (300 MHz, CDCl_3_, TMS): δ 1.03 (s, 18H,–6CH_3_), 1.27 (s, 18H,–6CH_3_), 3.16(d, 2H, *J* = 10.2 Hz, –2CH), 5.34(d, 2H, *J* = 10.4 Hz–2NH); ^13^C NMR (75 MHz, CDCl_3_): δ 23.38, 28.40, 37.15, 56.94, 65.55; IR (KBr, cm^-1^): 1040, 2869, 3192; *a* = -79.4 (c=0.94, CHCl3); HRMS for C_18_H_40_N_2_O_2_S_2_Na (M+Na): calcd. 403.2423, found: 403.2412.

### Refinement

The structure was solved by direct methods using SHELXS-97 and refined by full-matrix least-square calculation on F2 with SHELXL-97.

### Crystal data

**Table:**

C_18_H_40_N_2_O_2_S_2_	*D*_x_ = 1.107 Mg m^−^^3^
*M**_r_* = 380.64	Melting point: 408 K
Monoclinic, *P*2_1_/*c*	Mo *K*α radiation, λ = 0.71073 Å
*a* = 13.053 (2) Å	Cell parameters from 31 reflections
*b* = 9.578 (1) Å	θ = 2.7–12.9°
*c* = 18.279 (2) Å	µ = 0.25 mm^−^^1^
β = 92.069 (8)°	*T* = 287 K
*V* = 2283.6 (6) Å^3^	Block, colorless
*Z* = 4	0.50 × 0.44 × 0.38 mm
*F*(000) = 840	

### Data collection

**Table:**

Bruker P4 diffractometer	*R*_int_ = 0.014
Radiation source: normal-focus sealed tube	θ_max_ = 25.5°, θ_min_ = 1.6°
graphite	*h* = 0→15
ω scans	*k* = 0→11
4904 measured reflections	*l* = −22→22
4242 independent reflections	3 standard reflections every 97 reflections
3039 reflections with *I* > 2σ(*I*)	intensity decay: 4.6%

### Refinement

**Table:**

Refinement on *F*^2^	Secondary atom site location: difference Fourier map
Least-squares matrix: full	Hydrogen site location: inferred from neighbouring sites
*R*[*F*^2^ > 2σ(*F*^2^)] = 0.036	H atoms treated by a mixture of independent and constrained refinement
*wR*(*F*^2^) = 0.096	*w* = 1/[σ^2^(*F*_o_^2^) + (0.051*P*)^2^] where *P* = (*F*_o_^2^ + 2*F*_c_^2^)/3
*S* = 1.00	(Δ/σ)_max_ < 0.001
4242 reflections	Δρ_max_ = 0.20 e Å^−^^3^
238 parameters	Δρ_min_ = −0.15 e Å^−^^3^
2 restraints	Extinction correction: *SHELXL97* (Sheldrick, 2008), Fc^*^=kFc[1+0.001xFc^2^λ^3^/sin(2θ)]^-1/4^
Primary atom site location: structure-invariant direct methods	Extinction coefficient: 0.0048 (6)

### Special details

**Table:**

Geometry. All esds (except the esd in the dihedral angle between two l.s. planes) are estimated using the full covariance matrix. The cell esds are taken into account individually in the estimation of esds in distances, angles and torsion angles; correlations between esds in cell parameters are only used when they are defined by crystal symmetry. An approximate (isotropic) treatment of cell esds is used for estimating esds involving l.s. planes.
Refinement. Refinement of F^2^ against ALL reflections. The weighted R-factor wR and goodness of fit S are based on F^2^, conventional R-factors R are based on F, with F set to zero for negative F^2^. The threshold expression of F^2^ > 2sigma(F^2^) is used only for calculating R-factors(gt) etc. and is not relevant to the choice of reflections for refinement. R-factors based on F^2^ are statistically about twice as large as those based on F, and R- factors based on ALL data will be even larger.

### Fractional atomic coordinates and isotropic or equivalent isotropic displacement parameters (Å^2^)

**Table:**

	*x*	*y*	*z*	*U*_iso_*/*U*_eq_	
S1	0.09320 (4)	0.81382 (5)	0.57024 (2)	0.04452 (15)	
S2	0.41067 (4)	0.71457 (5)	0.64172 (2)	0.04755 (16)	
O1	0.16644 (10)	0.92962 (13)	0.55761 (8)	0.0603 (4)	
O2	0.33913 (10)	0.72124 (15)	0.70341 (7)	0.0616 (4)	
N1	0.15338 (11)	0.67388 (15)	0.60287 (8)	0.0393 (4)	
N2	0.34913 (11)	0.73713 (16)	0.56192 (8)	0.0405 (4)	
C1	0.02627 (15)	0.8664 (2)	0.65301 (11)	0.0514 (5)	
C2	−0.0445 (2)	0.7494 (3)	0.67459 (14)	0.0861 (8)	
H2A	−0.0045	0.6715	0.6923	0.103*	
H2B	−0.0860	0.7211	0.6328	0.103*	
H2C	−0.0879	0.7814	0.7124	0.103*	
C3	0.10290 (18)	0.9028 (3)	0.71364 (14)	0.0936 (9)	
H3A	0.0677	0.9410	0.7543	0.112*	
H3B	0.1505	0.9704	0.6962	0.112*	
H3C	0.1393	0.8202	0.7290	0.112*	
C4	−0.03526 (19)	0.9938 (3)	0.62925 (14)	0.0865 (8)	
H4A	−0.0817	0.9692	0.5894	0.104*	
H4B	0.0104	1.0656	0.6137	0.104*	
H4C	−0.0735	1.0272	0.6696	0.104*	
C5	0.19507 (13)	0.57327 (17)	0.55054 (9)	0.0385 (4)	
H5	0.1489	0.5749	0.5071	0.046*	
C6	0.18631 (14)	0.42373 (18)	0.58457 (11)	0.0472 (5)	
C7	0.24761 (17)	0.4111 (2)	0.65644 (12)	0.0705 (7)	
H7A	0.2234	0.4786	0.6906	0.085*	
H7B	0.3188	0.4278	0.6482	0.085*	
H7C	0.2394	0.3189	0.6760	0.085*	
C8	0.22317 (19)	0.3096 (2)	0.53305 (13)	0.0726 (7)	
H8A	0.2131	0.2197	0.5549	0.087*	
H8B	0.2947	0.3228	0.5246	0.087*	
H8C	0.1848	0.3147	0.4873	0.087*	
C9	0.07334 (16)	0.3948 (2)	0.59808 (14)	0.0714 (7)	
H9A	0.0663	0.3023	0.6175	0.086*	
H9B	0.0342	0.4024	0.5528	0.086*	
H9C	0.0487	0.4615	0.6325	0.086*	
C10	0.30488 (13)	0.61437 (18)	0.52394 (9)	0.0402 (4)	
H10	0.3503	0.5354	0.5357	0.048*	
C11	0.31135 (15)	0.6413 (2)	0.43952 (10)	0.0489 (5)	
C12	0.24953 (17)	0.7680 (2)	0.41434 (11)	0.0632 (6)	
H12A	0.2723	0.8486	0.4416	0.076*	
H12B	0.1782	0.7520	0.4223	0.076*	
H12C	0.2590	0.7836	0.3632	0.076*	
C13	0.27289 (18)	0.5154 (2)	0.39419 (11)	0.0713 (7)	
H13A	0.2012	0.5013	0.4018	0.086*	
H13B	0.3104	0.4335	0.4092	0.086*	
H13C	0.2830	0.5328	0.3432	0.086*	
C14	0.42397 (16)	0.6639 (3)	0.42250 (12)	0.0779 (7)	
H14A	0.4304	0.6734	0.3706	0.093*	
H14B	0.4637	0.5854	0.4397	0.093*	
H14C	0.4486	0.7472	0.4465	0.093*	
C15	0.48154 (15)	0.8818 (2)	0.64613 (11)	0.0545 (5)	
C16	0.55540 (17)	0.8832 (3)	0.58439 (13)	0.0728 (7)	
H16A	0.5176	0.8878	0.5384	0.087*	
H16B	0.5960	0.7996	0.5862	0.087*	
H16C	0.5995	0.9631	0.5894	0.087*	
C17	0.40897 (18)	1.0040 (3)	0.64227 (15)	0.0878 (8)	
H17A	0.3597	0.9949	0.6797	0.105*	
H17B	0.3741	1.0058	0.5951	0.105*	
H17C	0.4468	1.0891	0.6495	0.105*	
C18	0.5409 (2)	0.8768 (3)	0.71924 (13)	0.1062 (11)	
H18A	0.5844	0.9573	0.7239	0.127*	
H18B	0.5820	0.7937	0.7217	0.127*	
H18C	0.4936	0.8763	0.7583	0.127*	
H1N	0.1955 (11)	0.6939 (18)	0.6378 (7)	0.043 (5)*	
H2N	0.3093 (11)	0.8056 (13)	0.5610 (9)	0.033 (5)*	

### Atomic displacement parameters (Å^2^)

**Table:**

	*U*^11^	*U*^22^	*U*^33^	*U*^12^	*U*^13^	*U*^23^
S1	0.0499 (3)	0.0374 (3)	0.0460 (3)	0.0041 (2)	−0.0009 (2)	0.0020 (2)
S2	0.0482 (3)	0.0541 (3)	0.0397 (3)	−0.0101 (2)	−0.0082 (2)	0.0056 (2)
O1	0.0687 (9)	0.0382 (7)	0.0747 (10)	−0.0024 (7)	0.0123 (8)	0.0111 (7)
O2	0.0649 (9)	0.0805 (11)	0.0394 (7)	−0.0208 (8)	0.0021 (7)	0.0050 (7)
N1	0.0438 (9)	0.0358 (8)	0.0381 (8)	−0.0001 (7)	−0.0038 (7)	−0.0001 (7)
N2	0.0413 (9)	0.0408 (9)	0.0388 (8)	−0.0031 (8)	−0.0056 (7)	0.0021 (7)
C1	0.0544 (12)	0.0454 (11)	0.0546 (12)	0.0060 (10)	0.0054 (10)	−0.0059 (9)
C2	0.0939 (19)	0.0742 (17)	0.0929 (19)	−0.0062 (15)	0.0417 (16)	−0.0054 (14)
C3	0.0804 (17)	0.129 (2)	0.0717 (16)	0.0147 (17)	−0.0007 (14)	−0.0493 (16)
C4	0.0941 (19)	0.0639 (16)	0.103 (2)	0.0295 (14)	0.0211 (16)	−0.0018 (14)
C5	0.0426 (10)	0.0341 (9)	0.0383 (10)	−0.0013 (8)	−0.0069 (8)	−0.0015 (8)
C6	0.0508 (12)	0.0336 (10)	0.0567 (12)	−0.0027 (9)	−0.0049 (9)	0.0040 (9)
C7	0.0887 (17)	0.0508 (13)	0.0705 (15)	−0.0072 (12)	−0.0157 (13)	0.0220 (11)
C8	0.0924 (17)	0.0372 (12)	0.0882 (17)	0.0029 (12)	0.0042 (14)	−0.0030 (11)
C9	0.0670 (15)	0.0466 (13)	0.1006 (19)	−0.0140 (11)	0.0048 (13)	0.0095 (12)
C10	0.0447 (10)	0.0361 (10)	0.0392 (10)	0.0025 (8)	−0.0043 (8)	−0.0023 (8)
C11	0.0544 (12)	0.0545 (12)	0.0379 (10)	−0.0011 (10)	0.0016 (9)	−0.0047 (9)
C12	0.0832 (16)	0.0652 (14)	0.0410 (11)	−0.0007 (13)	0.0000 (11)	0.0088 (10)
C13	0.0964 (18)	0.0711 (15)	0.0459 (12)	−0.0033 (14)	−0.0028 (12)	−0.0128 (11)
C14	0.0707 (16)	0.111 (2)	0.0532 (13)	−0.0070 (15)	0.0197 (12)	−0.0089 (14)
C15	0.0534 (12)	0.0618 (13)	0.0478 (11)	−0.0208 (11)	−0.0056 (10)	−0.0025 (10)
C16	0.0651 (14)	0.0745 (16)	0.0795 (16)	−0.0209 (13)	0.0103 (13)	0.0034 (13)
C17	0.0820 (18)	0.0604 (16)	0.122 (2)	−0.0179 (14)	0.0155 (16)	−0.0283 (15)
C18	0.117 (2)	0.137 (3)	0.0618 (15)	−0.069 (2)	−0.0343 (15)	0.0106 (16)

### Geometric parameters (Å, °)

**Table:**

S1—O1	1.4877 (14)	C8—H8B	0.9600
S1—N1	1.6539 (15)	C8—H8C	0.9600
S1—C1	1.844 (2)	C9—H9A	0.9600
S2—O2	1.4913 (14)	C9—H9B	0.9600
S2—N2	1.6538 (15)	C9—H9C	0.9600
S2—C15	1.850 (2)	C10—C11	1.570 (2)
N1—C5	1.476 (2)	C10—H10	0.9800
N1—H1N	0.850 (9)	C11—C12	1.520 (3)
N2—C10	1.473 (2)	C11—C14	1.529 (3)
N2—H2N	0.837 (9)	C11—C13	1.537 (3)
C1—C3	1.507 (3)	C12—H12A	0.9600
C1—C2	1.513 (3)	C12—H12B	0.9600
C1—C4	1.516 (3)	C12—H12C	0.9600
C2—H2A	0.9600	C13—H13A	0.9600
C2—H2B	0.9600	C13—H13B	0.9600
C2—H2C	0.9600	C13—H13C	0.9600
C3—H3A	0.9600	C14—H14A	0.9600
C3—H3B	0.9600	C14—H14B	0.9600
C3—H3C	0.9600	C14—H14C	0.9600
C4—H4A	0.9600	C15—C17	1.505 (3)
C4—H4B	0.9600	C15—C16	1.511 (3)
C4—H4C	0.9600	C15—C18	1.521 (3)
C5—C6	1.567 (2)	C16—H16A	0.9600
C5—C10	1.580 (2)	C16—H16B	0.9600
C5—H5	0.9800	C16—H16C	0.9600
C6—C7	1.518 (3)	C17—H17A	0.9600
C6—C9	1.529 (3)	C17—H17B	0.9600
C6—C8	1.532 (3)	C17—H17C	0.9600
C7—H7A	0.9600	C18—H18A	0.9600
C7—H7B	0.9600	C18—H18B	0.9600
C7—H7C	0.9600	C18—H18C	0.9600
C8—H8A	0.9600		
			
O1—S1—N1	111.14 (8)	C6—C9—H9A	109.5
O1—S1—C1	104.49 (9)	C6—C9—H9B	109.5
N1—S1—C1	99.10 (8)	H9A—C9—H9B	109.5
O2—S2—N2	111.34 (8)	C6—C9—H9C	109.5
O2—S2—C15	104.82 (9)	H9A—C9—H9C	109.5
N2—S2—C15	98.71 (8)	H9B—C9—H9C	109.5
C5—N1—S1	118.48 (11)	N2—C10—C11	107.35 (14)
C5—N1—H1N	113.0 (12)	N2—C10—C5	113.49 (14)
S1—N1—H1N	112.0 (12)	C11—C10—C5	115.16 (14)
C10—N2—S2	118.77 (12)	N2—C10—H10	106.8
C10—N2—H2N	112.5 (12)	C11—C10—H10	106.8
S2—N2—H2N	113.7 (12)	C5—C10—H10	106.8
C3—C1—C2	112.0 (2)	C12—C11—C14	109.21 (18)
C3—C1—C4	110.80 (19)	C12—C11—C13	107.68 (16)
C2—C1—C4	110.38 (18)	C14—C11—C13	107.41 (18)
C3—C1—S1	110.19 (15)	C12—C11—C10	112.46 (15)
C2—C1—S1	108.89 (14)	C14—C11—C10	108.04 (16)
C4—C1—S1	104.26 (14)	C13—C11—C10	111.91 (16)
C1—C2—H2A	109.5	C11—C12—H12A	109.5
C1—C2—H2B	109.5	C11—C12—H12B	109.5
H2A—C2—H2B	109.5	H12A—C12—H12B	109.5
C1—C2—H2C	109.5	C11—C12—H12C	109.5
H2A—C2—H2C	109.5	H12A—C12—H12C	109.5
H2B—C2—H2C	109.5	H12B—C12—H12C	109.5
C1—C3—H3A	109.5	C11—C13—H13A	109.5
C1—C3—H3B	109.5	C11—C13—H13B	109.5
H3A—C3—H3B	109.5	H13A—C13—H13B	109.5
C1—C3—H3C	109.5	C11—C13—H13C	109.5
H3A—C3—H3C	109.5	H13A—C13—H13C	109.5
H3B—C3—H3C	109.5	H13B—C13—H13C	109.5
C1—C4—H4A	109.5	C11—C14—H14A	109.5
C1—C4—H4B	109.5	C11—C14—H14B	109.5
H4A—C4—H4B	109.5	H14A—C14—H14B	109.5
C1—C4—H4C	109.5	C11—C14—H14C	109.5
H4A—C4—H4C	109.5	H14A—C14—H14C	109.5
H4B—C4—H4C	109.5	H14B—C14—H14C	109.5
N1—C5—C6	107.79 (14)	C17—C15—C16	112.07 (19)
N1—C5—C10	113.38 (13)	C17—C15—C18	111.4 (2)
C6—C5—C10	115.46 (14)	C16—C15—C18	109.77 (18)
N1—C5—H5	106.5	C17—C15—S2	110.98 (14)
C6—C5—H5	106.5	C16—C15—S2	107.86 (15)
C10—C5—H5	106.5	C18—C15—S2	104.43 (15)
C7—C6—C9	109.08 (18)	C15—C16—H16A	109.5
C7—C6—C8	107.91 (17)	C15—C16—H16B	109.5
C9—C6—C8	107.22 (17)	H16A—C16—H16B	109.5
C7—C6—C5	111.88 (15)	C15—C16—H16C	109.5
C9—C6—C5	108.33 (16)	H16A—C16—H16C	109.5
C8—C6—C5	112.28 (16)	H16B—C16—H16C	109.5
C6—C7—H7A	109.5	C15—C17—H17A	109.5
C6—C7—H7B	109.5	C15—C17—H17B	109.5
H7A—C7—H7B	109.5	H17A—C17—H17B	109.5
C6—C7—H7C	109.5	C15—C17—H17C	109.5
H7A—C7—H7C	109.5	H17A—C17—H17C	109.5
H7B—C7—H7C	109.5	H17B—C17—H17C	109.5
C6—C8—H8A	109.5	C15—C18—H18A	109.5
C6—C8—H8B	109.5	C15—C18—H18B	109.5
H8A—C8—H8B	109.5	H18A—C18—H18B	109.5
C6—C8—H8C	109.5	C15—C18—H18C	109.5
H8A—C8—H8C	109.5	H18A—C18—H18C	109.5
H8B—C8—H8C	109.5	H18B—C18—H18C	109.5
			
O1—S1—N1—C5	−87.21 (14)	S2—N2—C10—C11	−147.20 (13)
C1—S1—N1—C5	163.31 (13)	S2—N2—C10—C5	84.38 (16)
O2—S2—N2—C10	−87.89 (15)	N1—C5—C10—N2	5.9 (2)
C15—S2—N2—C10	162.38 (14)	C6—C5—C10—N2	−119.21 (16)
O1—S1—C1—C3	−52.12 (18)	N1—C5—C10—C11	−118.43 (16)
N1—S1—C1—C3	62.63 (18)	C6—C5—C10—C11	116.50 (17)
O1—S1—C1—C2	−175.36 (15)	N2—C10—C11—C12	−62.0 (2)
N1—S1—C1—C2	−60.62 (17)	C5—C10—C11—C12	65.4 (2)
O1—S1—C1—C4	66.81 (16)	N2—C10—C11—C14	58.6 (2)
N1—S1—C1—C4	−178.44 (14)	C5—C10—C11—C14	−173.97 (16)
S1—N1—C5—C6	−146.44 (12)	N2—C10—C11—C13	176.62 (16)
S1—N1—C5—C10	84.47 (16)	C5—C10—C11—C13	−55.9 (2)
N1—C5—C6—C7	−61.1 (2)	O2—S2—C15—C17	−56.39 (17)
C10—C5—C6—C7	66.8 (2)	N2—S2—C15—C17	58.53 (17)
N1—C5—C6—C9	59.21 (19)	O2—S2—C15—C16	−179.51 (14)
C10—C5—C6—C9	−172.89 (16)	N2—S2—C15—C16	−64.59 (16)
N1—C5—C6—C8	177.42 (16)	O2—S2—C15—C18	63.76 (18)
C10—C5—C6—C8	−54.7 (2)	N2—S2—C15—C18	178.68 (17)

### Hydrogen-bond geometry (Å, °)

**Table:**

*D*—H···*A*	*D*—H	H···*A*	*D*···*A*	*D*—H···*A*
N1—H1N···O2	0.85 (1)	2.20 (1)	3.023 (2)	162 (2)
N2—H2N···O1	0.84 (1)	2.21 (1)	3.013 (2)	161 (2)
